# Choice of relative or cause-specific approach to cancer survival analysis impacts estimates differentially by cancer type, population, and application: evidence from a Canadian population-based cohort study

**DOI:** 10.1186/s12963-017-0142-4

**Published:** 2017-07-03

**Authors:** Diana R. Withrow, Jason D. Pole, E. Diane Nishri, Michael Tjepkema, Loraine D. Marrett

**Affiliations:** 1 Radiation Epidemiology Branch, National Cancer Institute, 9609 Medical Center Drive, Room 7E590, Bethesda, MD 20892-9778 USA; 2 Pediatric Oncology Group of Ontario, 480 University Avenue, Suite 1014, Toronto, ON M5G 1V2 Canada; 3 Population Health and Prevention, Prevention and Cancer Control, Cancer Care Ontario, 505 University Ave, 14th Floor, Toronto, ON M5G 1X3 Canada; 4 Health Analysis Division, Statistics Canada, 150 Tunney’s Pasture Driveway, Ottawa, ON K1A 0T6 Canada; 5 Aboriginal Cancer Control Unit, Prevention and Cancer Control, Cancer Care Ontario, 505 University Ave, 14th Floor, Toronto, ON M5G 1X3 Canada

**Keywords:** Neoplasms, Survival analysis, Minority health, Health status disparities, Indian, north American, Epidemiologic methods

## Abstract

**Background:**

Cause-specific (CS) and net survival in a relative survival framework (RS) are two of the most common methods for estimating cancer survival. In this paper, we assess the differences in results produced by two permutations of cause-specific and relative survival applied to estimating cancer survival and disparities in cancer survival, using data from First Nations and non-Aboriginal populations in Canada.

**Methods:**

Subjects were members of the 1991 Canadian Census Mortality Cohort, a population-based cohort of adult respondents to the 1991 Long Form Census who have been followed up for incident cancers and death through linkage to administrative databases. We compared four methods: relative survival analyses with ethnicity-specific life tables (RS-ELT); relative survival with general population life tables (RS-GLT); cause-specific survival with a broad definition of cancer death (CS-Broad); and cause-specific survival with a narrow definition of cause of death (CS-Narrow) and applied these to the nine most common cancers among First Nations.

**Results:**

Apart from breast and prostate cancers, RS-ELT, RS-GLT, and CS-Broad tended to produce similar estimates of age-standardized five-year survival, whereas CS-Narrow yielded higher estimates of survival. CS-Narrow estimates were particularly unlike those based on the other methods for cancers of the digestive and respiratory tracts. Estimates of disparities in survival were generally comparable across the four methods except for breast and prostate cancers.

**Conclusions:**

Cancer surveillance efforts in sub-populations defined by race, ethnicity, geography, socioeconomic status, or similar factors are necessary for identifying disparities and monitoring progress toward reducing them. In the absence of routine monitoring of cancer survival and cancer survival disparities in these populations, estimates generated by different methods will inevitably be compared over time and across populations. In this study, we demonstrate that caution should be exercised in making these comparisons, particularly in interpreting cause-specific survival rates with an unknown or narrow definition of cancer death and in estimates of breast and prostate cancer survival and/or disparities in survival generated by different methods.

**Electronic supplementary material:**

The online version of this article (doi:10.1186/s12963-017-0142-4) contains supplementary material, which is available to authorized users.

## Background

Cancer survival statistics are used to assess the quality of patient care and the effectiveness of new therapies, to monitor changes in prognosis over time, and to examine disparities among subgroups of the population, such as socioeconomic classes or ethnic groups. Most often, net survival is measured in these contexts – that is, the probability of survival from cancer in the absence of other causes of death. The two most common approaches for estimating net survival are relative survival and cause-specific survival [1]. The data available and the presumed validity of the data will often dictate which approach is used.

To estimate net survival in a relative survival framework, one takes a ratio of the observed survival of patients with cancer to the expected survival of a comparable group from the general population. The ratio is called a relative survival ratio. When relative survival is compared between populations, excess mortality rates are modeled and so the “relative” measure of relative survival is an excess mortality rate ratio. The major threat to validity for relative survival analysis is that the expected mortality (life tables) will not truly represent the counterfactual, cancer-free population [2–4].

Under a cause-specific framework, cancer deaths are considered events and patients who die from other causes are censored at their date of death. A 5-year cause-specific survival rate represents the proportion of patients who did not die of their cancer within 5 years of diagnosis. The “relative” measure of cause-specific survival is a hazard ratio. The major threat to validity for cause-specific survival analysis is misclassification of cause of death. Particularly for deaths caused by treatment side effects, among patients with multiple co-morbidities and as time since the initial cancer diagnosis increases, distinguishing and dichotomizing deaths as due to cancer or not may result in misclassification. For comparisons of survival between populations, this misclassification may be differential.

Because relative survival does not require a distinction or data about whether or not the death was attributable to cancer, and because of the fairly recent introduction of advanced statistical methods to measure and model relative survival and of easy-to-use software commands to implement them, relative survival has been promoted as the superior approach, especially for international comparisons of cancer survival [1, 5–8]. Meanwhile, a new, broader definition for cancer-specific deaths has been developed for estimating cause-specific survival that may be less prone to misclassification and that more closely approximates relative survival estimates [9].

Relative survival is the standard approach for cancer registry data but may not be suitable when appropriate life tables are not available, as is often the case when comparing survival between subsets of the population defined by ethnicity, social class, or smaller geographic regions, for example.

The few existing comparisons of estimates generated by cause-specific (CS) and relative survival (RS) methods have tended to be limited to the differences in RS and CS estimates in single populations [10–13]. Particularly for those investigators estimating differences in survival between populations for whom ideal life tables are not available, however, it is the difference in disparity measured by each method that is of greater interest than single population estimates. What may be small differences within a single population may be exaggerated when comparing survival across populations if the bias operates in opposite directions in the populations being compared.

In this paper, we measure cancer survival among non-Aboriginal Canadians and First Nation adults, and the disparities in cancer survival between these populations. Specifically, we compare estimates of 5-year survival rates and ratios estimated with (1) relative survival analyses using ethnicity-specific life tables; (2) relative survival analyses using life tables that are not ethnicity-specific; (3) cause-specific survival with a broad definition of cancer death; and (4) cause-specific survival with a narrow definition of cancer survival.

## Methods

### Data sources and methods

Subjects are members of the 1991 Census Mortality Cohort, a population-based cohort of adult respondents to the mandatory 1991 Canadian Long Form Census who have been followed up for incident cancers using probabilistic linkage to the Canadian Cancer Registry. Date and cause of death were ascertained using two sources: the Canadian Cancer Registry and the Canadian Mortality Database. Date of death was also supplemented with information from non-financial tax summary files. The Canadian Mortality Database includes all deaths occurring in Canada and deaths of Canadians occurring in some US states. A more thorough description of the cohort has been published elsewhere [14, 15].

We estimated survival from the nine cancers that were most common among First Nations. We restricted the study population to first primary cancers diagnosed in persons aged 45 to 90 between January 1, 2001, and December 31, 2009, excluding the province of Quebec. Quebec accounts for just under 25% of the Canadian population and about 10% of the First Nations population [16]. Cases from Quebec were excluded because of differences in registration procedures that make survival estimates non-comparable with those from other provinces/territories. We excluded cases identified based on death certificate or autopsy only or who had negative survival time (in total 1.1% of non-Aboriginal cases and 1.6% of First Nation cases). Cancers were grouped according to the Surveillance Epidemiology and End Results (SEER) site recodes based on the International Classification of Diseases for Oncology (ICD-O) 2nd and 3rd Editions conventions.

### Statistical analysis

We estimated 5-year age-standardized survival and excess mortality rate ratios/hazard ratios using four methods: relative survival with ethnicity-specific life tables (RS-ELT), relative survival with general population life tables (RS-GLT), cause-specific survival with a broad definition of cancer death (CS-Broad), and cause-specific survival with a narrow definition of cancer death (CS-Narrow). For all four methods, we used flexible parametric survival models, which employ restricted cubic splines and were implemented with the stpm2 command in Stata [8, 17]. Five-year survival was age-standardized to International Cancer Survival Standards, which vary by cancer type [18]. Age-standardized survival models were adjusted for sex if applicable. Excess mortality rate ratios and hazard ratios were stratified by sex and adjusted for age. All models used the complete approach, meaning that the conditional survival probabilities of all calendar years are used for all survival estimates [19]. More information about the models can be found in the Additional file 1.

Under both relative and cause-specific frameworks, survival time was the interval between the date of diagnosis and the date of death, 5 years post diagnosis, or December 31, 2009, whichever came first. For RS-ELT, expected survival was estimated using life tables produced based on the mortality experience of members of the cohort at large of the same age (single-year integers), sex (male, female), and ethnicity (First Nations or non-Aboriginal) during the same time period (1992–2000 and 2001–2009 rates were applied to each individual year within the time period) [20]. For RS-GLT, expected survival was estimated using life tables produced for the CONCORD-2 study [21]. These life tables were specific to age (single-year integers), sex (male, female), calendar year (single-year integers), and province/territory (nine provinces and three territories) but did not take ethnicity into account.

Under the narrow definition of cancer death (CS-Narrow) only deaths that were recorded as due to the first primary cancer were counted as events and all others were censored. The ICD-O codes used to produce this definition are available publicly from SEER [22]. The broad definition of cancer death (CS-Broad) was introduced by Howlader in 2010 and includes not only deaths attributed to the incident cancer directly, but also deaths attributable to other cancers, AIDS, and/or site-related diseases. What gets counted as a “cancer death” under the broad definition depends on whether the cancer was the only malignancy within an individual’s lifetime or the first of two or more malignancies and on the site of the of the original cancer diagnosis [9]. For both CS-Narrow and CS-Broad, if cause of death was missing (1.3% of non-Aboriginal cases and 2.3% of FN cases), patients were censored at their date of death.

## Results

Between January 1, 2001, and December 31, 2009, 82,805 cancers were diagnosed among non-Aboriginal cohort members and 1730 cancers were diagnosed among First Nation cohort members. As illustrated in Table 1, the crude proportion of cases that had died within 5 years of diagnosis was higher for First Nations than non-Aboriginals, despite First Nations having a median age at diagnosis that, for most cancers, was significantly lower than that among non-Aboriginal Canadians.

**Table 1:** Number of cases, deaths and median age at diagnosis, by cancer type and ethnicity^a^ (1991 Census Mortality Cohort 2001–2009)

	First Nations					Non-Aboriginal				
	Cases (n)	Deaths among cases within 5 years		Age at diagnosis		Cases (n)	Deaths among cases within 5 years		Age at diagnosis	
Cancer site		(n)	(%)	Median	IQR		(n)	(%)	Median	IQR
TOTAL^b^	1730	710	41.0	62.9	(55.4–70.9)	82,805	28,330	34.2	68.1	(59.8-76.3)
Colorectal	405	170	42.0	63.7	(55.8–72.6)	15,755	5665	36.0	70.8	(61.6-78.7)
Lung & bronchus	350	275	78.6	66.5	(59.3–73.7)	15,975	12,380	77.5	70.6	(62.7-77.6)
Breast	340	60	17.6	57.0	(50.9–64.7)	15,415	2020	13.1	62.3	(54.1-72.5)
Prostate	285	45	15.8	66.6	(60.6–73.6)	22,435	2835	12.6	68.4	(61.9-75.2)
Kidney	130	45	34.6	60.3	(53.9–69.7)	3150	1035	32.9	66.2	(57.6-75.0)
NHL	70	35	50.0	63.1	(54.5–72.5)	4840	1750	36.2	67.9	(58.6-77.0)
Stomach	60	45	75.0	59.6	(54.1–68.4)	2275	1610	70.8	71.6	(62.3-79.2)
Oral cavity & pharynx	45	20	44.4	61.6	(51.8–65.4)	2420	860	35.5	63.6	(55.8-73.4)
Cervix	35	15	42.9	59.6	(49.5–70.4)	545	170	31.2	56.6	(50.7-66.8)

*IQR* Interquartile range, *NHL* Non-Hodgkin lymphoma

^a^First primaries only, cases aged 45 to 90 at diagnosis, diagnosed between January 1, 2001, and December 31, 2009, excluding Quebec

^b^Counts have been randomly rounded to protect confidentiality as per Statistics Canada guidelines. Totals may not reflect the sum of the rounded counts

**Fig. 1:**
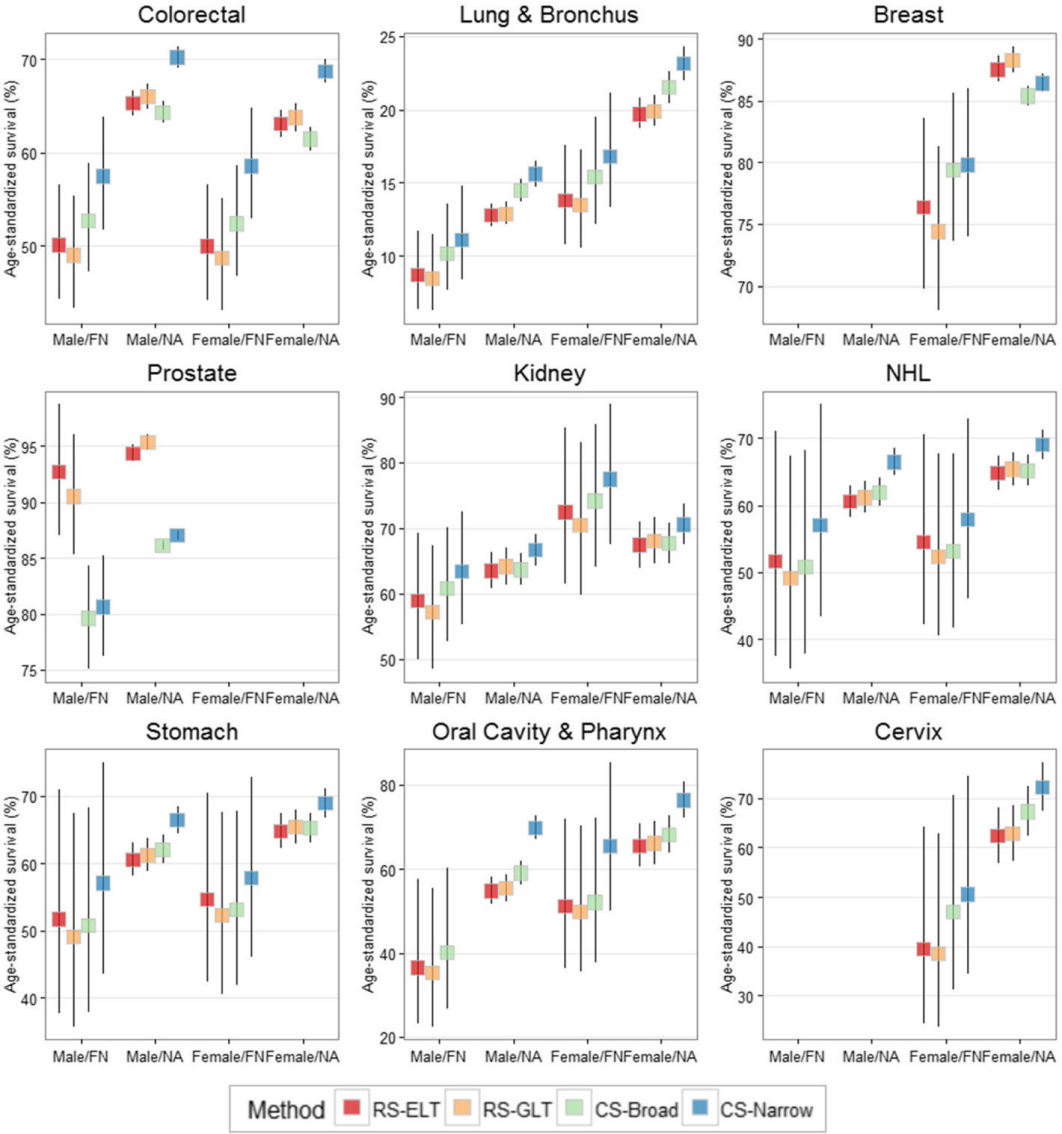
Age-standardized 5-year survival proportion by cancer, sex, ethnicity, and method. First primaries only, cases aged 45 to 90 at diagnosis, diagnosed between January 1, 2001, and December 31, 2009, excluding Quebec. Error bars indicate 95% confidence intervals. FN: First Nations; NA: Non-Aboriginal; NHL: Non-Hodgkin Lymphoma; RS-ELT: Relative Survival with ethnicity-specific life tables; RS-GLT: Relative Survival with general population life tables; CS-Broad: Cause-specific survival with a broad definition of cancer death; CS-Narrow: Cause-specific survival with a narrow definition of cancer death; NHL: Non-Hodgkin lymphoma

Age-standardized survival rates estimated using each of the four methods are displayed in Fig. 1. For all nine cancers included in our analyses, there was variation between the survival rates estimated by the four methods. While small numbers of cancers and deaths resulted in wide confidence intervals for survival within the First Nation population especially, point estimates for survival varied by nearly 10 percentage points for several cancer sites. (Refer to Additional file 1: Table S1).

Apart from breast and prostate cancers, CS-Narrow consistently overestimated survival compared to the other three methods. The largest relative differences were for stomach cancer (CS-Narrow was 2.11 times higher than RS-ELT for FN males), lung cancer (CS-Narrow was 1.17 to 1.28 times higher than RS-ELT), and cancers of the oral cavity and pharynx (CS-Narrow was 1.16 to 1.28 times higher than RS-ELT). The greatest differences between cause-specific and relative approaches were for breast and prostate cancer, where for all but breast cancer among First Nations, cause-specific survival was lower than relative survival irrespective of the cause of death definition used.

The RS-GLT approach underestimated age-standardized survival among First Nations compared to the RS-ELT approach, but the absolute differences between the RS-ELT and RS-GLT estimates never exceeded 3% and, given the wide confidence intervals, would be unlikely to alter interpretation. The RS-GLT approach slightly but consistently resulted in overestimates of survival among non-Aboriginals compared to RS-ELT. This is because the census-based cohort is somewhat healthier than the average Canadian, owing to a step in the probabilistic linkage that required cohort members to have filed taxes in 1990 or 1991 [15]. Using the background mortality of the entire Canadian population lowers the denominator (expected survival) and thereby inflates the non-Aboriginal survival ratio.

**Fig. 2:**
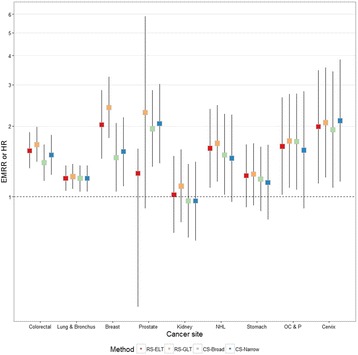
Five-year excess mortality rate ratios and hazard ratios for First Nations compared to non-Aboriginals. First primaries only, cases aged 45 to 90 at diagnosis, diagnosed between January 1, 2001, and December 31, 2009, excluding Quebec. Error bars indicate 95% confidence intervals. EMRR: Excess mortality rate ratio; HR: Hazard Ratio; RS-ELT: Relative Survival with ethnicity-specific life tables; RS-GLT: Relative Survival with general population life tables; CS-Broad: Cause-specific survival with a broad definition of cancer death; CS-Narrow: Cause-specific survival with a narrow definition of cancer death; NHL: Non-Hodgkin lymphoma; OC&P: Oral cavity & pharynx

Excess mortality rate ratios and hazard ratios estimated with each method are displayed in Table 2 and Fig. 2. Based on all four methods, First Nations with cancer had a survival deficit compared to their non-Aboriginal peers for all but kidney cancer (Fig. 2 and Table 2). The survival differential was statistically significant across all methods for colorectal, lung, breast, and cervical cancers. Compared to RS-ELT, RS-GLT produced slightly higher estimates of the deficit in survival associated with being First Nations, and CS-Narrow and CS-Broad produced slightly lower estimates.

**Table 2:** Five-year excess mortality rate ratios (EMRR) and hazard ratios (HR) and 95% confidence intervals (CI) for First Nations compared to non-Aboriginals, by method (1991 Census Mortality Cohort, 2001–2009)

	Relative survival with ethnicity-specific life tables		Relative survival with general population life tables		Cause-specific survival with a broad definition of cancer death		Cause-specific survival with a narrow definition of cancer death	
	(RS-ELT)		(RS-GLT)		(CS-Broad)		(CS-Narrow)	
Cancer site	EMRR	95% CI	EMRR	95% CI	HR	95% CI	HR	95% CI
Colorectal	**1.57**	(1.32–1.88)	**1.67**	(1.41–1.98)	**1.40**	(1.17–1.67)	**1.51**	(1.24–1.83)
Lung & bronchus	**1.20**	(1.06–1.36)	**1.22**	(1.08–1.38)	**1.20**	(1.05–1.36)	**1.20**	(1.05–1.36)
Breast	**2.03**	(1.45–2.84)	**2.40**	(1.78–3.24)	**1.47**	(1.05–2.06)	**1.56**	(1.11–2.18)
Prostate	1.26	(0.34–1.60)	2.29	(0.89–5.86)	**1.95**	(1.34–2.84)	**2.05**	(1.39–3.02)
Kidney	1.02	(0.70–1.49)	1.11	(0.78–1.59)	0.96	(0.67–1.38)	0.96	(0.65–1.41)
NHL	**1.61**	(1.09–2.37)	**1.69**	(1.16–2.46)	**1.51**	(1.02–2.25)	1.46	(0.95–2.24)
Stomach	1.23	(0.90–1.67)	1.25	(0.92–1.69)	1.19	(0.87–1.63)	1.15	(0.80–1.66)
Oral cavity & pharynx	**1.64**	(1.02–2.65)	**1.73**	(1.09–2.74)	**1.72**	(1.07–2.75)	1.58	(0.89–2.81)
Cervix	**1.99**	(1.14–3.46)	**2.07**	(1.21–3.55)	**1.93**	(1.09–3.42)	**2.11**	(1.16–3.84)

NOTES: Bolded text indicates a statistically significant disparity in survival between the two populations

First primaries only, cases aged 45 to 90 at diagnosis, diagnosed between January 1, 2001, and December 31, 2009, excluding Quebec

Adjusted for age and sex

The ranking of cancers from highest to lowest disparity varied depending on the method employed (not shown). This is partially attributable to chance – the number of cases and deaths among First Nations was relatively small for some cancers and the uncertainty around the effect estimates was high. This is also, however, attributable to a differential impact by cancer type of the biases associated with each method. The greatest absolute differences in the effect estimates generated by the four methods were for breast and prostate cancers.

## Discussion

Existing studies that have compared methods for estimating cancer survival have been focused on survival rates in single populations and have found that compatibility was poorest for rare cancers, as follow-up increases, and for cancers with high other-cause mortality [9–13]. We build on this literature by considering the implications of these methods for estimating cancer survival in specific subpopulations, here indigenous people, and for the estimation of disparities between subpopulations.

In brief, we found that for cancers of continuous organ systems (e.g., digestive and respiratory tract), cause-specific survival with a narrow definition of cause of death produced survival rates that differed significantly from those produced using the three other methods. Notably, a recent systematic review of studies of cancer survival among indigenous populations found that less than a third of nearly 50 included studies reported how they defined a cancer death [20]. With respect to disparities in cancer survival, estimates were fairly consistent across methods with the exception of breast and prostate cancers, for which the magnitude of disparity measured appeared to be dependent on the method employed.

### Cancer survival

Two major sources of bias will drive the differences in cancer survival between methods: (i) the extent to which the life tables represent the mortality of the cancer patients had they been cancer-free; and (ii) the proportion of cancer-consequent deaths that are attributed to cancer according to the death certificate and/or its interpretation [4]. For each cancer, the relative impact of these biases will vary.

In the case of prostate cancer, cause-specific survival was consistently lower than relative survival. This is consistent with other comparisons of relative and cause-specific survival for prostate cancer [9, 10, 12]. Cases were diagnosed in 2001 or later, by which time evidence had accumulated that prostate-specific antigen (PSA) testing should not be routinely used as a screening test [23, 24], but the PSA test may still have contributed to this difference. Higher uptake of the PSA test by people who are more socially advantaged and therefore healthier can result in underestimation of expected mortality in life tables and inflated estimates of relative survival from prostate cancer [25, 26].

Among non-Aboriginal women, estimates of RS from breast cancer were higher than estimates of CS survival. In contrast, among First Nation women, estimates of CS from breast cancer were higher than estimates of RS. American women with early-stage breast cancer have been shown to be less likely to die of non-cancer causes than other women of their same age without cancer [27]. Accordingly, if women with early-stage disease make up a large proportion of cases in the non-Aboriginal population, their expected mortality will be overestimated using general population life tables and the relative survival ratio will be inflated. First Nation women in Canada are less likely to be diagnosed with early stage disease, reducing the mismatch between life tables and true survival, and accordingly reducing the difference between survival measured by relative and cause-specific approaches [28].

For all cancers except prostate and breast, CS-Narrow produced higher survival estimates than the other three methods. This was expected – if cancer death is more narrowly defined, it will be rarer and cancer survival will appear higher. The differences in survival estimated by CS-Narrow compared to RS-ELT were greatest for stomach cancer, lung cancer, and cancers of the oral cavity and pharynx. The differences between the CS-Broad and CS-Narrow estimates for stomach cancer and cancers of the oral cavity and pharynx in particular suggest that the driving force behind these differences is a misclassification of cancer-consequent deaths as non-cancer under the narrow definition.

This was confirmed when we investigated the deaths counted as due to cancer under the broad definition but not under the narrow. For oral cavity and pharyngeal cancers, the discrepancy was mostly attributable to laryngeal cancer deaths that had not been counted as cancer deaths under the narrow definition because they were not of the same site. For stomach cancer, the discrepancy was a result of deaths attributed to esophageal cancer that had not been counted as cancer deaths under the narrow definition. These cancers stand out because cancers of the oral cavity, digestive tract, and respiratory tract are internal and contiguous, leading to a greater likelihood of attributing death to a neighboring organ.

Misattribution of cause of death along the continuous respiratory tract likely also explains, to some extent, the higher age-standardized survival from lung cancer calculated with CS-Narrow compared to the other methods. Unlike stomach and oral cavity and pharyngeal cancers, however, the CS-Broad estimate was also inflated compared to the RS-approaches. This suggests that the relative survival approach may be underestimating survival. Since lung cancer is highly associated with smoking, and smoking is associated with poorer health and more comorbidities, general population life tables overestimate expected survival among lung cancer patients [27].

### Disparities in cancer survival

Differences across methods in the relative risk measures will only arise if the assumptions underlying each method are violated to different extents in the subgroups being compared. For example, the use of general population life tables rather than ethnicity-specific ones introduces a differential bias. General population life tables underestimate the background mortality of First Nations to a greater extent than they overestimate the background mortality of non-Aboriginals. In this study, for all but breast and prostate cancers, this bias had a minimal effect on the EMRRs, which were comparable under RS-ELT and RS-GLT frameworks.

For prostate cancer, cause-specific approaches produced HRs that indicated a significant difference in survival that was not found with relative survival. The cause-specific HRs for prostate cancer were lower than the EMRRs estimated by RS-GLT but higher than the EMRRs estimated by RS-ELT. The biases we have previously described do not clearly explain this pattern; however there is substantial uncertainty around these estimates because of the small proportion of cases that died during the 5-year follow-up, so the pattern we see may be due to chance.

For breast cancer, CS estimates of disparity were lower than RS estimates. As described above, this is likely attributable at least in part to a differential bias introduced by life tables (even ethnicity-specific ones) for First Nations and non-Aboriginal Canadians. In the general population, where breast cancers may more often be screen-detected, life tables would underestimate expected survival due to the “healthy screener” effect [29, 30]. First Nation women, in contrast, may be more likely to be diagnosed incidentally while in contact with the health system for other illnesses/comorbidities, in which case life tables would overestimate expected survival. More research to explore methods of detection and how they vary in Indigenous and non-Indigenous persons would be worthwhile to further explore this hypothesis.

### Limitations and generalizability

For many cancers, there were small numbers of cases and deaths among First Nations. The resulting uncertainty around survival estimates for First Nations could have led to an under-ascertainment of what, with more power, may be statistically significant differences between the estimates using different methods. We therefore considered the differences in interpretation of effect estimates produced by each of the approaches rather than the statistical relationship between them. A second consequence of the small numbers was an inability to consider differences between the methods with longer follow-up or by age. Based on existing literature, we would expect the differences between the methods to be greater with increasing time since diagnosis and among older patients [11–13].

While the small numbers may not have permitted such analyses, stage-specific comparisons, particularly for breast and prostate cancers, would provide insight into the factors driving differences between estimates. Stage has not been historically collected in Canadian cancer registries and so was not available for analysis in this study.

The analyses presented in this paper were limited to first primary cancers because the broad definition of cancer death is only applicable to first cancers [9]. Given that the incidence of second cancers is rising, and that second cancers are accounting for a growing proportion of all cancers, we believe that including higher-order malignancies in survival estimates is generally good practice. The development of a broad classification of cancer-specific death for higher-order cancers would be valuable.

We used a model-based approach to estimate net survival in a relative survival framework. The relatively new Pohar Perme estimator of net survival is an alternative approach that has been recently introduced and promoted. The Pohar Perme estimator is theoretically unbiased because it is not subject to the influence of informative censoring mechanisms whereby the people who are least likely to die overall (and therefore would presumably have the highest cancer survival) contribute more time into the expected mortality denominator [31]. The disadvantages to the Pohar Perme estimator are that it is non-parametric and has high variability. The high variability is exaggerated if relative survival ratios are age-standardized [31]. Given the small numbers of cancers and deaths among First Nations in this study, and the similarities in estimates produced by model-based methods and the Pohar Perme estimator for short follow-up times, such as the 5-year time horizon in this study, we think the model-based approach was justified.

With respect to generalizability, the magnitude of the differences in results generated by each method will be affected by the differences between the populations being compared. If, for example, uptake of breast cancer screening was similar in both populations, we might expect to see smaller differences in the measures of disparity. Furthermore, the dataset used for this study had a high level of completeness of cause of death data. In general, the exclusion of persons with missing cause of death from cause-specific analyses, while they are included in the relative survival estimates, could introduce further differences between the estimates generated by each method.

## Conclusions

Relative survival has been advocated for use in international studies where cause of death may be classified differently across jurisdictions. Given the potential for differential misclassification of cause of death by ethnicity or socioeconomic status, the same argument may apply to comparisons of survival across subgroups within a population [32]. Our results show that for estimates of 5-year cancer survival in a single population, the biases associated with each of the approaches acts to different extents depending on the cancer and the population. For seven of the nine cancers, a cause-specific approach with a narrow definition of cause of death leads to higher estimates of cancer survival compared to the other methods.

When survival in these two populations was compared using an excess mortality rate ratio or hazard ratio, the biases that resulted in differing survival for single population estimates tended to cancel each other out, yielding comparable estimates of disparity irrespective of the approach used. Breast and prostate cancers, however, two of the most common cancers, were exceptions. For these cancers, estimates of disparities varied meaningfully depending on the analytic approach.

Cancer surveillance in sub-populations defined by race, ethnicity, geography, socioeconomic status, and other similar factors are crucial to identifying disparities and monitoring progress toward reducing them. RS-ELT and CS-Broad approaches are preferable over RS-GLT and CS-Narrow approaches, respectively, but, particularly in these types of populations, are not always used [20]. In the absence of routine surveillance, results of studies that have used different methods will inevitably be compared to monitor survival and disparities between populations and over time. To what degree should caution be exercised in making these comparisons? In this study, we demonstrate that cause-specific survival measured using a narrow definition of cancer death does not generally yield estimates comparable to the other methods. Furthermore, cause-specific and relative survival approaches generally are not comparable for estimates of survival or disparity in survival from breast and prostate cancers.

## Additional file

Additional file 1: Supplementary Material: Detailed description of life tables and stpm2 models and table displaying excess mortality rate ratios (EMRRs) and hazard ratios (HR) for First Nations compared to non-Aboriginals by method, sex, and ethnicity. (DOCX 28 kb)
