# Supplementary material for: Choice of relative or cause-specific approach to cancer survival analysis impacts estimates differentially by cancer type, population, and application: evidence from a Canadian population-based cohort study
Source: Popul Health Metr. 2017 Jul 3;15:24. doi: 10.1186/s12963-017-0142-4 (PMC5496357; doi:10.1186/s12963-017-0142-4)
Supplement: Supplementary file 1 — Supplementary Material: Detailed description of life tables and stpm2 models and table displaying excess mortality rate ratios (EMRRs) and hazard ratios (HR) for First Nations compared to non-Aboriginals by method, sex, and ethnicity. (DOCX 28 kb) [file 12963_2017_142_MOESM1_ESM.docx]

**SUPPLEMENTARY MATERIAL**

1. **Life tables and stpm2 models**

Life tables for the RS-ELT method were constructed using flexible parametric models implemented with the stpm2 command in Stata version 13(1). Given the relatively large amount of random variation in the observed mortality rates at either end of the age spectrum for First Nations, we restricted our life tables to ages 45 to 90 years. Within this age range, mortality rates were more precise and therefore deviation of the models from empirical rates could be identified and model fit improved. Calendar time was dichotomized at the midpoint of follow-up time (i.e., 1992-2000 and 2001-2009) because finer splitting of calendar time would have yielded models based on unstable calendar-year-specific rates for First Nations.

The flexible parametric models were employed to produce life tables, to estimate survival rates and to estimate excess mortality rate ratios. For each model, knots were tested in non-default positions and when appropriate, we included interaction terms between the spline terms and the covariates of interest to take into account non-proportionality. In the survival models, for each cancer site we tested for time-varying effects of age (in 10 year groups from 45 until 85-90), sex, and ethnicity, where the primary time scale was the time since diagnosis. For all models, fit was assessed based on a combination of Akaike’s Information Criterion, Bayes Information Criterion, log likelihood ratio tests and subjective judgment of graphical displays since simple use of AIC and BIC can occasionally lead to over-fitting.(2)

References

1. Lambert P. STPM2: Stata module to estimate flexible parametric survival models. Statistical Software Components. Boston College Department of Economics; 2010.

2. Royston P, Lambert PC. Flexible Parametric Survival Analysis Using Stata. Stata Press; 2011.

1. **Supplementary Table 1**

| **Table S1.** Excess mortality rate ratios (EMRRs) and hazard ratios (HR) for First Nations compared to non-Aboriginals by method, sex, and ethnicity. | | | | | | | | | | | | |
| --- | --- | --- | --- | --- | --- | --- | --- | --- | --- | --- | --- | --- |
|  |  | | |  | | |  | | |  | | |
|  | **RS-ELT** | | | **RS-GLT** | | | **CS-Broad** | | | **CS-Narrow** | | |
| **Cancer site** | **EMRR**  **(%)** | 95% upper bound | CI lower bound | **EMRR**  **(%)** | 95% upper bound | CI lower bound | **HR**  **(%)** | 95% upper bound | CI lower bound | **HR**  **(%)** | 95% upper bound | CI lower bound |
|  |  |  |  |  |  |  |  |  |  |  |  |  |
| **MALES - FIRST NATIONS** | | | |  |  |  |  |  |  |  |  |  |
| Colorectal | 50.1 | 44.3 | 56.6 | 49.0 | 43.4 | 55.4 | 52.7 | 47.2 | 58.9 | 57.5 | 51.8 | 63.9 |
| Lung & Bronchus | 8.7 | 6.4 | 11.7 | 8.5 | 6.3 | 11.5 | 10.2 | 7.7 | 13.6 | 11.1 | 8.4 | 14.8 |
| Prostate | 92.7 | 87.0 | 98.7 | 90.5 | 85.3 | 96.0 | 79.6 | 75.1 | 84.3 | 80.6 | 76.2 | 85.2 |
| Kidney | 58.9 | 50.0 | 69.3 | 57.2 | 48.6 | 67.4 | 60.9 | 52.8 | 70.2 | 63.4 | 55.4 | 72.6 |
| NHL | 51.7 | 37.6 | 71.0 | 49.1 | 35.7 | 67.4 | 50.8 | 37.9 | 68.2 | 57.1 | 43.5 | 75.0 |
| Stomach | 15.6 | 8.6 | 28.1 | 15.3 | 8.5 | 27.6 | 17.7 | 10.1 | 31.0 | 32.9 | 21.4 | 50.6 |
| Oral cavity & pharynx° | 36.5 | 23.2 | 57.5 | 35.4 | 22.5 | 55.6 | 40.2 | 26.8 | 60.4 |  |  |  |
| **MALES - NON-ABORIGINALS** | | |  |  |  |  |  |  |  |  |  |  |
| Colorectal | 65.3 | 64.0 | 66.6 | 66.0 | 64.7 | 67.4 | 64.3 | 63.2 | 65.5 | 70.2 | 69.1 | 71.4 |
| Lung & Bronchus | 12.8 | 12.0 | 13.6 | 12.9 | 12.2 | 13.7 | 14.5 | 13.7 | 15.3 | 15.6 | 14.7 | 16.5 |
| Prostate | 94.3 | 93.6 | 95.1 | 95.3 | 94.6 | 96.0 | 86.1 | 85.7 | 86.5 | 87.0 | 86.6 | 87.4 |
| Kidney | 63.5 | 60.8 | 66.4 | 64.2 | 61.4 | 67.1 | 63.7 | 61.3 | 66.2 | 66.7 | 64.3 | 69.2 |
| NHL | 60.6 | 58.2 | 63.0 | 61.2 | 58.9 | 63.7 | 62.0 | 60.0 | 64.2 | 66.4 | 64.4 | 68.5 |
| Stomach | 22.2 | 19.6 | 25.0 | 22.4 | 19.8 | 25.2 | 23.5 | 21.0 | 26.2 | 38.0 | 34.7 | 41.5 |
| Oral cavity & pharynx | 54.8 | 51.7 | 58.2 | 55.4 | 52.2 | 58.8 | 59.1 | 56.3 | 62.0 | 69.8 | 67.1 | 72.6 |
| **FEMALES - FIRST NATIONS** | | | | |  |  |  |  |  |  |  |  |
| Colorectal | 50.0 | 44.2 | 56.6 | 48.7 | 43.1 | 55.1 | 52.4 | 46.8 | 58.6 | 58.6 | 52.9 | 64.8 |
| Lung & Bronchus | 13.8 | 10.8 | 17.6 | 13.5 | 10.6 | 17.3 | 15.4 | 12.2 | 19.5 | 16.8 | 13.3 | 21.1 |
| Breast | 76.4 | 69.8 | 83.6 | 74.4 | 68.0 | 81.3 | 79.4 | 73.6 | 85.6 | 79.8 | 74.0 | 86.0 |
| Kidney | 72.5 | 61.5 | 85.3 | 70.5 | 59.9 | 83.1 | 74.2 | 64.1 | 85.8 | 77.5 | 67.5 | 88.9 |
| NHL | 54.6 | 42.3 | 70.5 | 52.3 | 40.5 | 67.6 | 53.2 | 41.8 | 67.7 | 57.9 | 46.1 | 72.8 |
| Stomach | 21.0 | 13.2 | 33.4 | 20.8 | 13.1 | 33.0 | 22.9 | 14.7 | 35.6 | 29.9 | 19.7 | 45.3 |
| Oral cavity & pharynx | 51.1 | 36.4 | 71.9 | 49.9 | 35.5 | 70.2 | 52.1 | 37.7 | 72.2 | 65.4 | 50.2 | 85.3 |
| Cervix | 39.5 | 24.2 | 64.3 | 38.6 | 23.7 | 62.8 | 47.0 | 31.3 | 70.7 | 50.6 | 34.3 | 74.6 |
| **FEMALES - NON-ABORIGINALS** | | | | |  |  |  |  |  |  |  |  |
| Colorectal | 63.1 | 61.7 | 64.6 | 63.8 | 62.3 | 65.3 | 61.5 | 60.2 | 62.8 | 68.7 | 67.5 | 70.0 |
| Lung & Bronchus | 19.7 | 18.7 | 20.8 | 19.9 | 18.9 | 21.0 | 21.5 | 20.4 | 22.6 | 23.1 | 22.0 | 24.3 |
| Breast | 87.5 | 86.5 | 88.6 | 88.3 | 87.3 | 89.4 | 85.4 | 84.6 | 86.2 | 86.4 | 85.7 | 87.2 |
| Kidney | 67.5 | 64.0 | 71.1 | 68.1 | 64.7 | 71.8 | 67.7 | 64.7 | 70.9 | 70.6 | 67.5 | 73.7 |
| NHL | 64.8 | 62.3 | 67.4 | 65.4 | 62.9 | 67.9 | 65.2 | 63.0 | 67.5 | 69.0 | 66.8 | 71.2 |
| Stomach | 27.4 | 23.6 | 31.8 | 27.7 | 23.8 | 32.1 | 28.0 | 24.4 | 32.2 | 34.1 | 30.0 | 38.8 |
| Oral cavity & pharynx | 65.5 | 60.6 | 70.8 | 66.1 | 61.2 | 71.4 | 68.2 | 63.9 | 72.8 | 76.3 | 72.2 | 80.7 |
| Cervix | 62.4 | 57.0 | 68.2 | 62.8 | 57.4 | 68.7 | 67.3 | 62.5 | 72.5 | 72.3 | 67.5 | 77.3 |
| ° Estimates for CS-Narrow among First Nation males not releasable under Statistics Canada data release guidelines. RS-ELT: Relative Survival with ethnicity-specific life tables; RS-GLT: Relative Survival with general population life tables; CS-Broad: Cause-specific survival with a broad definition of cancer death; CS-Narrow: Cause-specific survival with a narrow definition of cancer death; NHL: Non-Hodgkin lymphoma. | | | | | | | | | | | | |
